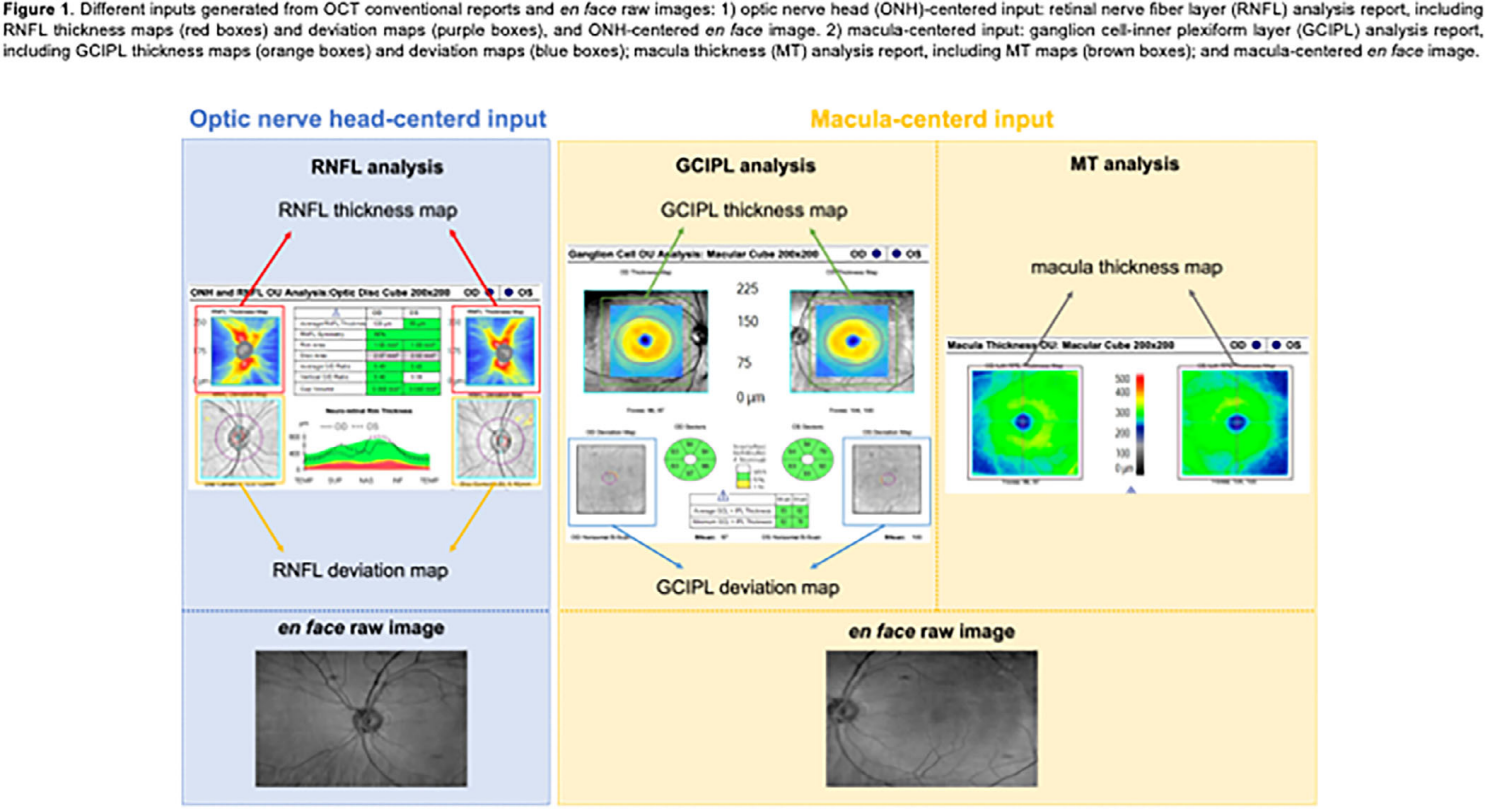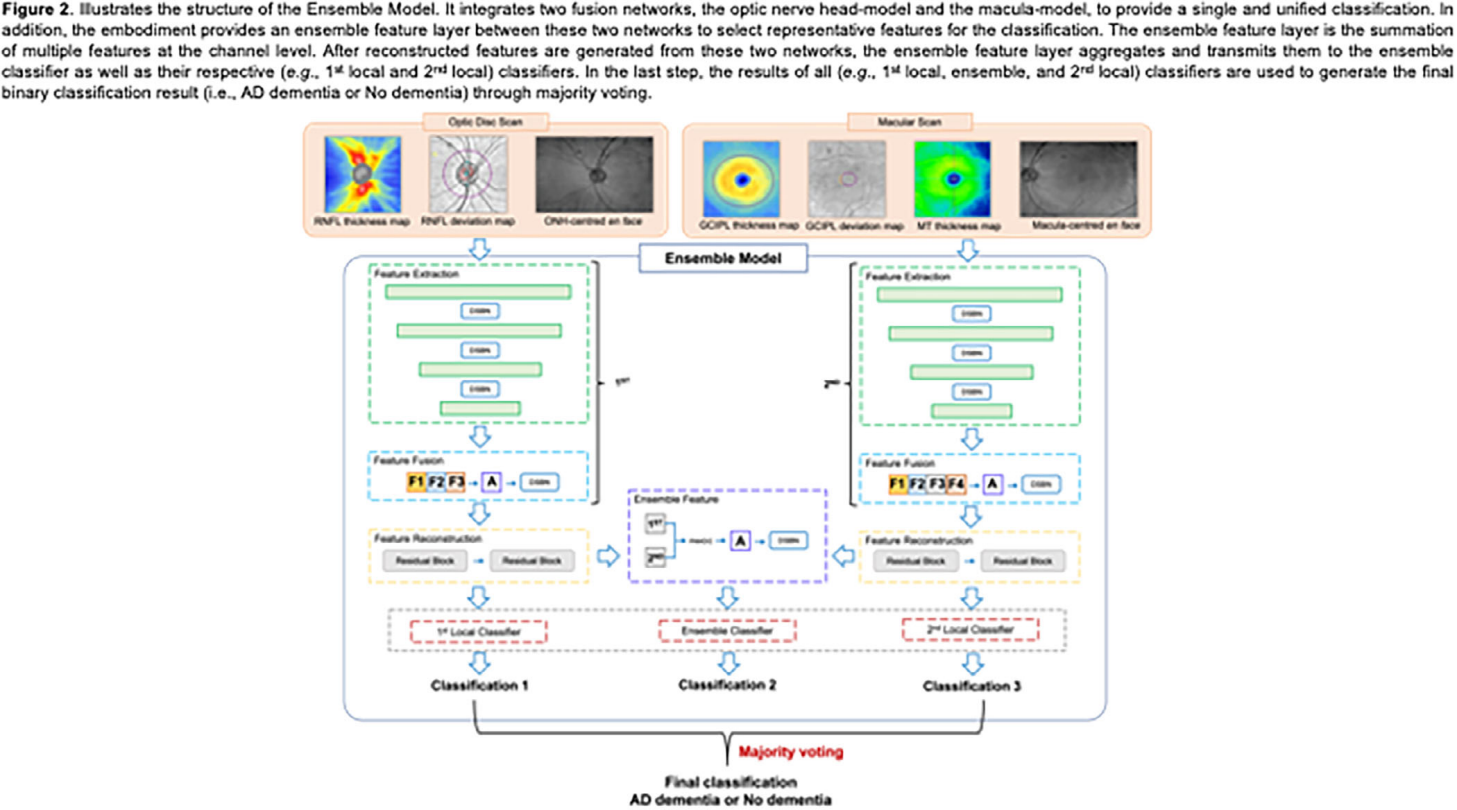# Applying FusionNet and Ensemble Learning‐based Artificial Intelligence Techniques for Alzheimer's Disease Detection from Retinal Optical Coherence Tomography

**DOI:** 10.1002/alz70856_100619

**Published:** 2025-12-25

**Authors:** Anran Ran, Herbert Y.H. Hui, Xiaoyan Hu, Christopher Li‐Hsian Chen, Tien Yin Wong, Vincent C.T. Mok, Carol Y Cheung

**Affiliations:** ^1^ Department of Ophthalmology and Visual Sciences, The Chinese University of Hong Kong, Hong Kong SAR, NA, Hong Kong; ^2^ Lam Kin Chung. Jet King‐Shing Ho Glaucoma Treatment and Research Centre, The Chinese University of Hong Kong, Hong Kong SAR, Hong Kong; ^3^ The Chinese University of Hong Kong, Hong Kong, NA, Hong Kong; ^4^ Memory Ageing and Cognition Centre, National University Health System, Singapore, NA, Singapore; ^5^ Department of Pharmacology, Clinical Research Centre, National University Health System, National University of Singapore, Singapore, Singapore; ^6^ Yong Loo Lin School of Medicine, National University of Singapore, Singapore, Singapore; ^7^ National University Hospital, Kent Ridge, Singapore; ^8^ Tsinghua Medicine, Tsinghua University, Beijing, NA, China; ^9^ The Chinese University of Hong Kong, Shatin, Hong Kong

## Abstract

**Background:**

Nearly half of Alzheimer's dementia could be prevented or delayed simply by addressing some modifiable risk factors. We aim to develop novel deep‐learning (DL) models with fusion network and ensemble learning techniques for an automated detection of Alzheimer's Disease (AD) using retinal optical coherence tomography (OCT) to facilitate AD identification for timely intervention.

**Method:**

We trained, validated, and tested DL models for classifying AD‐dementia or no dementia using *en face* images and analysis reports of OCT (Cirrus HD‐OCT device, Carl Zeiss Medite, Inc., Dublin, CA). Specifically, the optic nerve head (ONH)‐ and macula‐centred *en face* images and three OCT conventional analysis reports were used, namely (1) retinal nerve fibre layer (RNFL) analysis, (2) macular thickness (MT) analysis, and (3) ganglion cell‐inner plexiform layer (GCIPL) analysis. The RNFL and GCIPL analysis reports contain thickness and deviation maps, while the MT report only contains the thickness map. To analyze multiple inputs from a single eye for AD‐dementia detection, we proposed a fusion network, including “Feature Extraction”, “Feature Fusion”, and “Feature Reconstruction”. We developed fusion network‐based DL models, including the ONH Model, Macula Model, and Integrated Model, using different inputs of OCT. We further incorporated the ensemble learning technique to integrate all inputs into a single model (i.e., Ensemble Model).

**Result:**

For model training and internal validation, we retrospectively collected 3,228 paired OCT reports and *en face* images from 1,239 subjects with AD‐dementia and 1,800 cognitively normal controls. For model external testing, we retrospectively collected 86 paired OCT reports and *en face* images from 38 subjects in Hong Kong as External‐1, and 125 paired OCT reports and *en face* images from 60 subjects in Singapore as External‐2. In the internal validation, External‐1, and External‐2, the ONH Model achieved accuracies of 85.4%, 76.3%, and 73.0%, respectively. The Macula Model achieved accuracies of 82.4%, 76.5%, and 70.7%, respectively. The Integrated Model achieved accuracies of 84.8%, 74.1%, and 71.7%, respectively. The Ensemble Model achieved accuracies of 90.5%, 80.3%, and 74.2%, respectively.

**Conclusion:**

Our proposed Ensemble Model that learned AD‐related retinal features from OCT analysis shows a large potential to identify individuals with AD‐dementia accurately.